# Protective Effect of *Meso*-Tetrakis-(3,5-di-*tert*-butyl-4-hydroxyphenyl)porphyrin on the In Vivo Impact of Trimethyltin Chloride on the Antioxidative Defense System

**DOI:** 10.1155/BCA/2006/64927

**Published:** 2006-12-27

**Authors:** Elena R. Milaeva, Vladimir Yu. Tyurin, Yulia A. Gracheva, Margarita A. Dodochova, Lydia M. Pustovalova, Victor N. Chernyshev

**Affiliations:** ^1^Department of Organic Chemistry, M. V. Lomonosov Moscow State University, Moscow 119992, Russia; ^2^Department of Biochemistry, Rostov State Medicinal University, Rostov on Don 344022, Russia

## Abstract

The in vivo effect of trimethyltin chloride (Me_3_SnCl), free base *meso*-tetrakis(3,5-di-*tert*-butyl-4-hydroxyphenyl)porphyrin (R′_4_PH_2_) and their equimolar mixture, on the enzymatic activity of catalase (CAT), superoxide dismutase (SOD), and on the total content of free sulfhydryl groups has been studied in rat liver and kidney. It was demonstrated that the simultaneous treatment of tested animals with the combination of Me_3_SnCl and R′_4_PH_2_ reduced the toxic impact of Me_3_SnCl.

## INTRODUCTION

Organotin compounds find a considerably application in industry and agriculture and the subsequent discharge of toxic organotins into the environment is a topic of great concern [1, 2]. Among the organotins R_n_SnX_4−n_ trimethyltin species Me_n_SnX_4−n_ are of particular importance since these compounds are formed in the environment in biomethylation processes [3].

The toxicity of organotins is associated with their ability to react with free SH-groups in proteins and glutathione and to inhibit the activities of some enzymes.

On the other hand, it is well known that organotins induce oxidative stress in the living organism through multiple mechanisms including the enhancement of the intracellular generation of reactive oxygen species (ROS), H_2_O_2_, O^−•^_2_, HO^•^ [4]. The involvement of R_n_SnX_4−n_ in radical and redox biochemical processes is manifested in C−Sn bond homolytic cleavage that leads to the generation of reactive C-centered organic radicals R^•^ [5]. Thus a very reactive methyl radical CH^•^_3_ might be formed when methyl derivatives of tin, Me_n_SnX_4−n_, participate in biochemical radical reactions. The consequences of this impact are the perturbation of the antioxidative defense system and the promotion of a cascade of radical processes. On the other hand, the metal ions formed in the biodegradation of organotins are promoters of radical processes.

Therefore, an intriguing aspect of the behavior of organotins R_n_SnX_4−n_ is their ability to manifest the activity of metal containing prooxidants and chain radical reactions promoters as well.

The disruption of the oxidative status in the living organism can be prevented or inhibited by cellular antioxidants. Toxic doses of organotin compounds are capable of disturbing the natural oxidation/reduction balance in cells through various mechanisms originating from their own complex oxidative/radical reactions with endogenous oxidants. The consequences of these reactions produce some effects on cellular antioxidant systems, cellular membranes, and membrane-dependent redox sensitive enzymatic systems. This, in turn, may produce a variety of toxic effects, including pathological processes, which lead to the cells death.

Therefore, there is an urgent need to find new detoxification agents to prevent or inhibit the disruption of the cellular antioxidative system when organotins are involved.

Processes caused by active radical species are prevented or inhibited by treating the organism with natural or synthetic antioxidants. The application of chelating agents such as metal scavengers seems to be important in order to exclude the impact of the metal ion.

Lately, a new approach has been proposed [6] to prevent the prooxidative activity of organotins by applying a specific polytopic compound capable of acting as an antioxidant and a metal ion scavenger—free base *meso*-tetrakis(3,5-di-*tert*-butyl-4-hydroxyphenyl)porphyrin, R′_4_PH_2_. This exogenous compound acts as an inhibitor since its molecules contain the antioxidative phenol moieties, analogues of vitamins E group. The other pathway of R′_4_PH_2_ is associated with the ability of free base porphyrins to incorporate metal ions in their core and to form stable metal complexes.

The influence of Me_2_SnCl_2_, Et_2_SnCl_2_, and SnCl_2_ upon the radical chain oxidation of Z-9-octadecenoic (oleic) acid as model substrate for lipid peroxidation in the simultaneous presence of R′_4_PH_2_ has been studied [6]. The free base porphyrin R′_4_PH_2_, containing the antioxidative phenol moieties (2,6-di-*tert*-butylphenol), demonstrates an acute inhibitory effect upon the oleic acid's peroxidation in the presence of organotins.

Thus we suppose that *meso*-tetrakis(3,5-di-*tert*-butyl-4-hydroxyphenyl)porphyrin can act as an antioxidant and a scavenger for metal and can be used as a new antioxidative scavenger preventing the toxic impact of organotin compounds.

The goal of the present study is to evaluate the in vivo protective effect of *meso*-tetrakis(3,5-di-*tert*-butyl-4-hydroxyphenyl)porphyrin against the impact of Me_3_SnCl upon the components of antioxidative defense system (catalase and superoxide dismutase) using rats as tested organisms. The total level of SH-groups as a marker of Me_3_SnCl impact upon proteins containing thiol groups and glutathione in rats' organs has been studied as well.

## EXPERIMENTAL

### Materials and instruments

The following materials were obtained commercially and used as supplied: Me_3_SnCl (Strem), (NH_4_)_6_MO_7_O_24_, 5,5′-dithio-bis(2-nitrobenzoic) acid (DTNB), nitroblue tetrazolium (NBT), EDTA (Sigma). Free base *meso*-tetrakis(3,5-di-*tert*-butyl-4-hydroxyphenyl)porphyrin was synthesized as previously described by the known procedure [7], purified by silica gel column chromatography using CHCl_3_, 80% CHCl_3_ and 20% hexane as the eluting solvents, and identified by UV-vis and IR spectroscopy. Deionized water purified with Simplicity Proto system (Millipore) was used. The solutions of Me_3_SnCl were prepared by dissolving the precise quantity of the compound in Tween-80. The solutions of Me_3_SnCl were prepared directly before the analysis. Spectrophotometric study was performed by using spectrophotometer SF-46 (LOMO, Russia) and Varian 100S spectrophotometer. Infrared (IR) spectra were recorded on a Perkin Elmer “Spectrum One” spectrophotometer.

### Experimental animals

Adult female Wistar rats (200–220 g body wt) were used throughout experiments. The animals were divided into 4 groups (a control group of animals that did not receive any treatment, three experimental groups of animals which received either Me_3_SnCl or *meso*-tetrakis(3,5-di-*tert*-butyl-4-hydroxyphenyl)porphyrin or their combination, resp; each group contained 5 animals). The animals were given a single oral additive's dose of 5 mg·kg body wt. This is a less amount of Me_3_SnCl than the oral LD_50_-dose of about 9 mg·kg [8]. The simultaneous oral treatment of the animals with Me_3_SnCl and *meso*-tetrakis(3,5-di-*tert*-butyl-4-hydroxyphenyl)porphyrin was performed in an analogous way by using the same doses. The rats were clearly affected by the treatment (diminished mobility, anxiety, aggression). However, none of the tested animals died during the experiment. The animals were killed after 24 h, the livers and kidneys were immediately removed, rinsed with ice-cold saline, homogenized in 0.05 M phosphate buffer (pH 7.4) and 0.1 mM EDTA using a motor-driven Teflon-glass homogenizer followed by ultrasonification as described previously [9]. After centrifugation at 2000× g for 10 min, the supernatant was used for the analysis.

### Measurement of catalase and superoxide dismutase activities and the content of glutathione

The liver and kidney tissues were used for measurement of catalase (CAT) (EC 1.11.1.6) and superoxide dismutase (SOD) (EC 1.15.1.1) activities as well as for measuring the content of free sulfhydryl groups.

The assay for the determination of catalase activity was carried out as described previously [10, 11] by a method based on the disappearance of H_2_O_2_ in the reaction of hydrogen peroxide with (NH_4_)_6_MO_7_O_24_ monitored spectrophotometrically at 410 nm.

The assay for the determination of SOD activity was carried out as described previously [12] by a method using nitroblue tetrazolium (NBT) as the indicator reagent spectrophotometrically at 560 nm [11].

The content of SH-groups was measured by the reaction of free sulfhydryl groups with 5,5′-dithio-bis(2-nitrobenzoic) acid (DTNB) spectrophotometrically at 412 nm as described previously [13]. In all the experiments, Tween-80 impact upon the activity of CAT, SOD, and the content of SH-groups has been preliminary studied. No significant changes in the activities of enzymes and of the content of SH-groups have been observed.

### Statistical analysis

**Figure 1:**
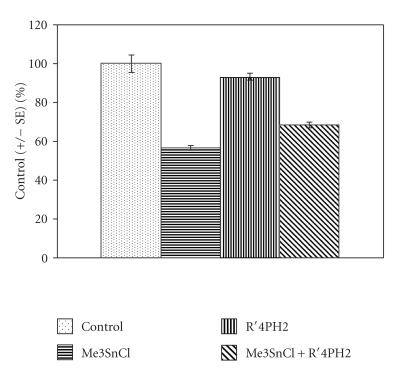
Effect of Me_3_SnCl, R′_4_PH_2_, and their combination exposure on the activity of CAT in rat liver (rat liver was isolated after 24 h after the animals were pretreated orally with 5 mg·kg^−1^ wt of Me_3_SnCl, R′_4_PH_2_, and their combination, resp; *P* < .05).

**Figure 2:**
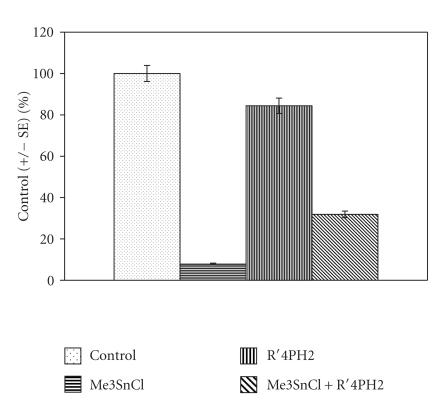
Effect of Me_3_SnCl, R′_4_PH_2_, and their combination exposure on the activity of SOD in rat liver (rat liver was isolated after 24 h after the animals were pretreated orally with 5 mg·kg^−1^ wt of Me_3_SnCl, R′_4_PH_2_, and their combination, resp; *P* < .05).

**Figure 3:**
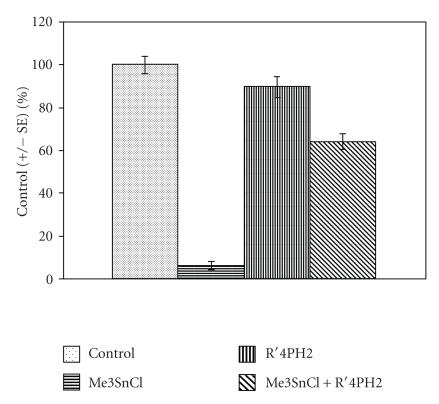
Effect of Me_3_SnCl, R′_4_PH_2_, and their combination exposure on SH-groups content in rat liver (rat liver was isolated after 24 h after the animals were pretreated orally with 5 mg·kg^−1^ wt of Me_3_SnCl, R′_4_PH_2_, and their combination, resp; *P* < .05).

All the data displayed in Table 1 and Figures 1, 2, and 3 are presented as means of several experiments ± standard errors (SE) and represent treatment-induced changes. The assays of enzymes activities and free sulfhydryl groups' content were carried out in 8 or 12 parallel experiments. The significance of differences between experimental conditions was tested at the 5% level (*P* < .05). The Kolmogorov-Smirnov test was used to assess the normality of the distribution of each treatment [14].

## RESULTS AND DISCUSSION

Organic derivatives of tin (R_n_SnX_m_) are supposed to induce oxidative stress in the living organism through multiple mechanisms including the intracellular generation of reactive oxygen species (ROS) [1, 3, 4, 15–17], depletion of SH-groups in proteins and glutathione, promotion of lipid peroxidation, and perturbation of antioxidant defense system [4]. To understand the biomolecular mode of organotin compounds action, the participation of various R_n_SnX_m_ in key biochemical processes responsible for the damage of the antioxidative defense system should be further studied.

The involvement of R_n_SnX_m_ in oxidative/free radical reactions may include the reactions of these compounds with very reactive radical species. These processes lead to the homolytic cleavage of C−Sn bond and result in the formation of reactive organic radicals R^•^ responsible for the enhanced perturbation of the antioxidative defense system and cell death [5]. Therefore, there is a need to elaborate a new approach in order to prevent or inhibit the impact of R_n_SnX_4−n_ upon the complex antioxidative defense system.

Methyl derivative of Sn(IV) possessing three methyl groups (Me_3_SnCl) was selected for the investigation since there was strong evidence that this compound is the dominant species presented in biota [1, 3].

**Scheme 1:**
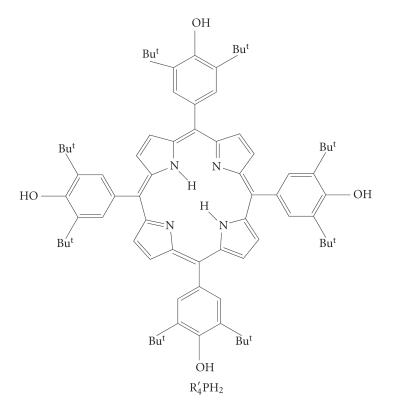


A new efficient route to prevent the prooxidative activity of the organotin compounds was proposed and based on free base porphyrin containing antioxidative phenolic groups (*meso*-tetrakis(3,5-di-*tert*-butyl-4-hydroxyphenyl)porphyrin) [6] (see Scheme 1).

It was established that this compound acts as an effective antioxidant inhibiting the peroxidation of oleic acid in the presence of organotins [18]. Moreover, free base porphyrins are capable of incorporating metal ions in their core [19] when these macrocyclic compounds are involved in oxidative/radical processes.

### Effect of trimethyltin chloride and *meso*-tetrakis(3,5-di-*tert*-butyl-4-hydroxyphenyl)porphyrin upon the activity of catalase

Both organic and inorganic tins have been found to decrease the activity of catalase—an H_2_O_2_ scavenger [20, 21]. The mechanism of the enzyme inhibition is associated with the interaction of Sn center with free SH-groups in protein. Trimethyltin chloride is capable of interacting with SH-groups according to the nucleophilic substitution reaction of chlorine atom at metal center. On the other hand, the inorganic tin formed in the dealkylation of Me_3_SnCl in radical substitution reactions may also interact readily with SH-groups. Therefore the protective effect of R′_4_PH_2_ might be of importance since both centers in its molecule are responsible for both radical processes inhibition and metal ion scavenging.

The influence of these substances upon the enzyme activity in rat liver and kidney was studied. The data for enzymes activity and free sulfhydryl groups content are presented in Table 1 and given in Figure 1 as percent of control (± SE).

It was shown that the catalytic activity of CAT significantly decreased when the animals were treated orally with the dose of Me_3_SnCl 5 mg·kg^−1^ wt. At the same time it was observed that the same amount of R′_4_PH_2_ had almost no effect on CAT activity, whereas Me_3_SnCl at the same concentration inhibited the enzyme sufficiently. The treatment of the tested animals with the combination of both additives is manifested in the significant decrease in Me_3_SnCl impact upon the enzyme.

### Effect of trimethyltin chloride and *meso*-tetrakis(3,5-di-*tert*-butyl-4-hydroxyphenyl)porphyrin upon the activity of superoxide dismutase

Superoxide dismutase (SOD) is involved in the functioning of cellular antioxidative system and is responsible for the dismutation of highly toxic superoxide radical anion O2^•−^ in cells. The inhibition of this antioxidant enzyme activity by metals is a well-known fact [22]. The inhibition of SOD is discussed as one of the mechanisms of organotins cytotoxicity as well [2, 4, 5].

The data are presented in Table 1 and Figure 2 for both isolated liver and kidney tissues when rats were treated with the dose of Me_3_SnCl 5 mg·kg^−1^ wt. The results are analogous to the previous ones presenting the effect of additives on the CAT activity. The equal dose of R′_4_PH_2_ does not show any significant effect on SOD activity, whereas the treatment of rats with the mixture of both compounds is manifested in significant modulation of Me_3_SnCl impact upon the enzymatic activity.

### Effect of trimethyltin chloride and *meso*-tetrakis(3,5-di-*tert*-butyl-4-hydroxyphenyl)porphyrin upon the content of free sulfhydryl groups

In the present study the content of free SH-groups has been examined as a biomarker of the protective effect of R′_4_PH_2_ against the toxic impact of Me_3_SnCl. The results are given in Table 1 and Figure 3. The protective effect of porphyrin R′_4_PH_2_ for both experimental tissues—liver and kidney—isolated after the treatment of rats is very significant. These experimental results suggest that the interaction of Me_3_SnCl (or of inorganic tin as the product of Me_3_SnCl decomposition) may be prevented by the application of R′_4_PH_2_.

Thus, it was demonstrated that trimethyltin chloride causes the inhibition of CAT and SOD catalytic activities that can be explained in terms of possible coupling with SH-groups of the enzymes. The decrease in the content of free sulfhydryl groups in rat tissues was observed as well.

Recently, we have studied the influence of methyltins (MeSnCl_3_, Me_2_SnCl_2_, and Me_3_SnCl), and inorganic tins (SnCl_2_, SnCl_4_) on the enzymatic activities of NAD-dependent horse liver alcohol dehydrogenase (ADH) in the reaction of ethanol oxidation [23] and NAD-dependent lactate dehydrogenase isolated from fish liver [24]. The results show that inorganic tins and organotins induce inhibition of the catalytic activity of horse liver alcohol dehydrogenase. It also turned out that the mechanism of methyltins action is more complex than the proposed interaction of Sn with SH-groups of the enzyme protein. It was clearly demonstrated that the tin compounds act as oxidative agents towards coenzyme NADH as well.

Therefore, the results allow one to suggest that both mechanisms (coupling with the SH-groups in proteins and involvement in oxidative/radical processes) might be responsible for the impact of Me_3_SnCl upon the cellular antioxidative enzymatic system. The decrease of Me_3_SnCl effect in the simultaneous presence of R′_4_PH_2_ confirms the assumption that this polytopic compound might act as a radical and metal scavenger.

## CONCLUSION

In summary, the presented experimental results of this in vivo study show that trimethyltin chloride induces the inhibition of the catalytic activities of CAT and SOD in rat liver and kidney and decreases the level of free SH-groups as well. It was demonstrated that the simultaneous treatment of tested rats with free base *meso*-tetrakis(3,5-di-*tert*-butyl-4-hydroxyphenyl)porphyrin significantly attenuates Me_3_SnCl toxic impact. Taken together, our results suggest that porphyrin containing antioxidative phenol fragments might act as a radical and metal scavenger.

## Figures and Tables

**Table 1:** Activities of catalase, superoxide dismutase, and total content of SH-groups in rat liver and kidney.*

	Enzyme activity, mmol·mg^−1^ · min^−1^				Total content of SH-groups, mmol·mg^−1^	
	
	CAT		SOD			
	
	Liver	Kidney	Liver	Kidney	Liver	Kidney

Control	241.0 ± 11.0	157.6 ± 5.8	12.8 ± 0.5	6.7 ± 0.2	155.4 ± 6.4	136.3 ± 6.5
Me_3_SnCl	136.5 ± 6.7	102.1 ± 3.8	1.0 ± 0.05	0.5 ± 0.01	9.5 ± 0.5	32.8 ± 0.8
Me_3_SnCl + R′_4_PH_2_	164.8 ± 6.4	128.2 ± 1.9	3.8 ± 0.06	2.1 ± 0.1	99.4 ± 3.8	62.6 ± 3.2
R′_4_PH_2_	224.8 ± 8.4	144.9 ± 4.3	10.8 ± 0.4	6.6 ± 0.3	139.1 ± 6.9	132.1 ± 0.5

* After 24 h of oral treatment of rats with both trimethyltin chloride and porphyrin R′_4_PH_2_ each alone as well as in combination (*P* < .05 versus control). Data ± SE (*n* = 8 − 12); *n* = number of replicates of enzyme activity measurements.
